# ﻿Afrotropical Dirrhopinae (Hymenoptera, Braconidae) with description of three new species

**DOI:** 10.3897/zookeys.1259.172473

**Published:** 2025-11-12

**Authors:** Beibei Zheng, Zeqiu Xian, Zhen Liu, Andrew Polaszek

**Affiliations:** 1 Zoology Key Laboratory of Hunan Higher Education, College of Life and Environmental Sciences, Hunan University of Arts and Science, Changde 415000, China; 2 Hunan Provincial Key Laboratory for Health Aquaculture and Product Processing in Dongting Lake Area, Hunan University of Arts and Science, Changde 415000, China; 3 Science: Research, Natural History Museum, London, SW7 5BD, UK

**Keywords:** *
Dirrhope
*, identification key, Madagascar, new taxon, South Africa, taxonomy

## Abstract

The rare braconid subfamily Dirrhopinae van Achterberg is formally recorded for the first time from the Afrotropical region. Three new species of *Dirrhope* Foerster: *D. albobasalis* Liu & Polaszek, **sp. nov.**, *D. nixoni* Liu & Polaszek, **sp. nov.** and *D. ocellimacula* Liu & Polaszek, **sp. nov.** are described, and an illustrated key to these species is provided.

## ﻿Introduction

Dirrhopinae (Hymenoptera, Braconidae) are extremely rare, with only six species known in the sole genus *Dirrhope* Foerster (Yu et al. 2016; Chen & van Achterberg 2019; Ranjith et al. 2021). *Dirrhope* has had a complicated taxonomic history, having been included in Microgastrinae (Foerster 1863; Muesebeck 1935; Shenefelt 1973; Marsh 1979), Miracinae (Tobias 1986; Belokobylskij 1989) and Adeliinae (Telenga 1955; Capek 1970) before it was confirmed as the only valid genus in the subfamily Dirrhopinae. It is considered to be closely related to the microgastroid complex of subfamilies based on morphological data (van Achterberg 1984; Quicke and van Achterberg 1990; Wharton et al. 1992; Whitfield and Mason 1994; Belokobylskij 1998; Belokobylskij et al. 2003). More recently, molecular data supported it as the sister subfamily to Cheloninae within the microgastroid complex (Jasso-Martínez et al. 2023).

Dirrhopinae, currently including species distributed in the Australian, Nearctic, Oriental and Palaearctic regions, was recently revised by Ranjith et al. (2021). The Afrotropical region, however, has been overlooked until now, although Nixon (1965) stated that some specimens in the British Museum (now Natural History Museum, London) agreed well with the interpretation of *Dirrhope* by Muesebeck (1935). Here, we describe three new species (two from South Africa and one from Madagascar) from the London Natural History Museum collection.

## ﻿Material and methods

Specimens studied are deposited in the Natural History Museum, UK (**NHMUK**). Descriptions and measurements were made using a stereomicroscope (Zeiss® Stemi SV6). Photographs were taken and processed using a digital camera (Zeiss AxioZoom or Hirox HRX-01) combined with Helicon Focus software. The images were further processed using Adobe Photoshop® CS6. Morphological terms for body structures and measurements follow Belokobylskij (1989) and Ranjith et al. (2021). The wing vein terminology follows the modified Comstock-Needham system (van Achterberg 1993). The terminology of the cuticular sculpture follows Harris (1979). Abbreviations used are as follows: **POL** = postocellar line, **OOL** = ocular-ocellar line, **OD** = ocellar diameter; **T1** = 1^st^ tergite of metasoma, **T2** = 2^nd^ tergite of metasoma, **T3** = 3^rd^ tergite of metasoma.

## ﻿Taxonomy

### ﻿Genus Dirrhope

Taxon classificationAnimaliaHymenopteraBraconidae

Foerster

B50BFEE7-EEC2-561F-927A-29E766EDA106

Dirrhope Foerster, 1851: 39. Type species: Dirrhope rufa Foerster.
Dirrhope Foerster: Muesebeck 1935: 173; Telenga 1955: 14; Tobias 1986: 459; Capek 1970: 870; Shenefelt 1973: 675; Marsh 1979: 256; Belokobylskij 1998: 547; Wu et al. 2000: 203; Ranjith et al. 2021: 251.

#### Diagnosis.

Head and mesosoma rather robust, granulate or densely reticulate-rugose, metasoma comparatively weak; head transverse; occipital carina absent or incomplete; eye glabrous; frons concave laterally, distinctly protruding medially, with one longitudinal ridge-like carina extending to area between antennal sockets, sometimes to middle of face; antenna with 18–24 antennomeres, first flagellomere slightly longer than second; anterior tentorial pits relatively large; clypeus convex, protruding in lateral view; clypeal suture deep; malar suture present; maxillary palp with 6 palpomeres, third palpomere distinctly swollen; labial palp with 4 palpomeres; propleural lobe present; notauli usually incomplete, distinct basally; prepectal carina present; precoxal sulcus present but shallow; propodeum distinctly areolate and carinate; fore wing vein 1-R1 present; vein r-m of fore wing absent; vein 2-1A largely obsolescent; second submarginal and subdiscal cells open; veins 1-M and 1-SR+M arising from parastigma; vein m-cu antefurcal; vein cu-a distinctly postfurcal; and first tergite relatively slender, parallel-sided, spiracles situated behind middle of first tergite (see Ranjith et al. 2021).

##### ﻿Key to species of *Dirrhope* from the Afrotropical region

**Table:** 

1	Propodeum with sublateral cells near areola (Fig. 1e); mesoscutum with punctures strongly confluent to strigose posteriorly (Fig. 1h); basal three to four flagellomeres white (Fig. 1d)	***D. albobasalis* Liu & Polaszek, sp. nov.**
–	Propodeum without sublateral cells near areola (Figs 2i, 3g); mesoscutum not strigose posteriorly (Figs 2e, 3f); flagellomeres evenly yellow-brown (Figs 2d, 3b)	**2**
2	Antenna 18-merous (Fig. 2d); midlongitudinal carina 1.3 × length of its apical bifurcated carina on propodeum (Fig. 2i); notauli distinct at anterior declivity, absent posteriorly (Fig. 2e)	***D. nixoni* Liu & Polaszek, sp. nov.**
–	Antenna 19-merous (Fig. 3b); midlongitudinal carina 0.9 × length of its apical bifurcated carina on propodeum (Fig. 3g); notauli extending to scutellar sulcus (Fig. 3f)	***D. ocellimacula* Liu & Polaszek, sp. nov.**

Note. Females of *D. albobasalis* Liu & Polaszek, sp. nov. are unknown for this study.

### ﻿ Dirrhope albobasalis

Taxon classificationAnimaliaHymenopteraBraconidae

Liu & Polaszek sp. nov.

2F3DFD1B-1A18-5600-9C5F-A57501111DBE

https://zoobank.org/911EB88C-3707-4C4B-B8F0-874C47D2FB6D

Fig. 1

#### Diagnosis.

**Figure 1.**
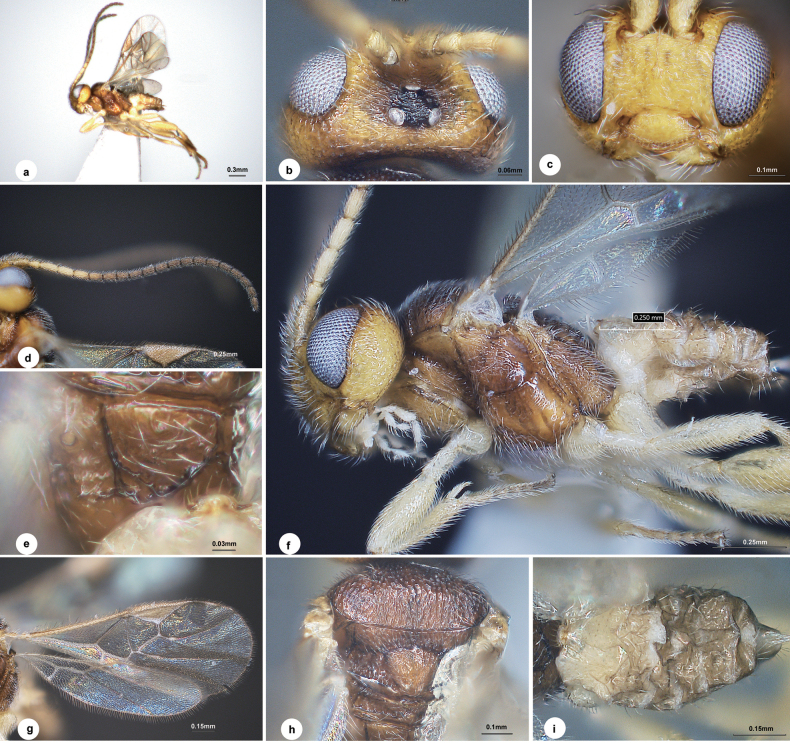
*Dirrhope albobasalis* Liu & Polaszek, sp. nov., male. a. Habitus, lateral aspect; b. Head, dorsal aspect; c. Head, anterior aspect; d. Antenna; e. Propodeum; f. Head, mesosoma and metasoma, lateral aspect; g. Wings; h. Mesosoma, dorsal aspect; i. Metasoma, dorsal aspect.

Body length 1.7 mm, mesosoma brown, head yellow-brown to black on stemmaticum, and metasoma white to light brown (Fig. 1a); eyes (Fig. 1b) 1.3 × longer than temple in dorsal view; vertex and frons with numerous transverse curved carinae; temple weakly coarsely rugose, distinctly bulging behind eyes in dorsal view; POL:OD:OOL = 1.4:1.0:2.9; median carina on face distinctly loop-shaped at end; face (Fig. 1c) weakly strigose mostly, 1.4 × wider than high; antenna (Fig. 1d) distinctly longer than body length, 20-merous, with penultimate flagellomere 1.4 × longer than wide, closely articulated; mesoscutum (Fig. 1h) with punctures confluent to strigose posteriorly, notauli distinct at anterior declivity, absent posteriorly; scutellar sulcus wide with ill-defined carinae inside; scutellar hind depressions large and oblong; propodeum (Fig. 1e) with midlongitudinal carina half the length of its apical bifurcated carina, sublateral cells present; mesopleuron (Fig. 1f) weakly rugulose medially and rugose above; pterostigma (Fig. 1g) 3.0 × as long as its maximum width; vein 1-R1 0.8 × length of pterostigma; vein r 0.6 × of maximum width of pterostigma, 0.5 × 2-SR; sclerotised part of 3-SR 0.7 × as long as r; vein m-cu 1.3 × as long as 2-SR+M; hind femur 2.8 × as long as its maximum width; inner spur of hind tibia half as long as hind basitarsus; T1 narrow, smooth, hardly visible in dorsal view; T2 and following tergites weakly sclerotised, smooth (Fig. 1i).

#### Description.

**Male.** Body length 1.7 mm, fore wing length 1.4 mm (Fig. 1a).

***Head*.** 1.9 × as wide as long, 1.1 × wider than mesoscutum. Eyes 1.3 × longer than temple in dorsal view (Fig. 1b). Vertex and frons slightly shiny with numerous transversely curved carinae. Temple weakly coarsely rugose, distinctly bulging behind eyes in dorsal view. Ocelli small, distance between fore and a hind ocellus 1.2 × longer than minor axis of a hind ocellus, POL:OD:OOL = 1.4:1.0:2.9. Frons flat with a weak median carina passing between antenna sockets to middle of face, carina distinctly loop-shaped at end. Face (Fig. 1c) shiny, strigose, transverse, 1.4 × wider than high. Clypeus 1.8 × wider than medial length, weakly rugulose laterally. Length of malar space 0.8 × width of mandible. Antenna (Fig. 1d) distinctly longer than body length, 20-merous, with scape, pedicel and 1^st^, 2^nd^, penultimate and ultimate flagellomeres 1.8, 1.3, 2.3, 2.2, 1.4 and 1.9 × longer than wide, 1^st^ indistinctly longer than 2^nd^, flagellomeres gradually shortened to penultimate flagellomere, closely articulated.

***Mesosoma*.** Length:width:height = 1.4:1.0:1.0. Mesoscutum (Fig. 1h) shiny, with even punctures anteriorly which are confluent to strigose posteriorly, notauli distinct at anterior declivity, absent posteriorly. Scutellar sulcus wide with ill-defined carinae inside. Scutellum shiny, rugulose-punctate, hind depressions large and oblong, separated by a carina. Propodeum (Fig. 1e) less shiny, basally shallowly punctate with one midlongitudinal carina half the length of its apical bifurcated carina, a broad pentagonal areola present posteriorly, sublateral cells attached to areola anterior-laterally, remaining parts uneven. Mesopleuron (Fig. 1f) shiny, weakly rugulose medially and rugose above. Prepectal carina distinct.

***Wings*.** Fore wing (Fig. 1g): pterostigma wide, 3.0 × as long as its maximum width; vein 1-R1 0.8 length of pterostigma; vein r arising from middle of pterostigma, 0.6 × of maximum width of pterostigma, 0.5 × 2-SR; sclerotised part of 3-SR 0.7 × as long as r; vein m-cu 1.3 × as long as 2-SR+M, 0.4 × 2-SR; vein 1-CU1 about as long as cu-a. Hind wing (Fig. 1g): vein M+CU:1-M:r-m = 3.1:2.0:1.0.

***Legs*.** Hind femur 2.8 × as long as its maximum width. Hind tibia distinctly widened apically, its length 3.5 × maximum width, as long as hind femur. Inner spur of hind tibia half the length of hind basitarsus. Hind basitarsus 0.7 × as long as tarsomeres 2–5.

***Metasoma*.** 0.8 × length of mesosoma. T1 (Fig. 1i) narrow, smooth, hardly visible in dorsal view. T2 and following tergites distinctly desclerotised, smooth, suture between T2 and T3 indistinct. Hypopygium not exceeding apex of metasoma.

***Colour*.** Mesosoma brown, head yellow-brown to slightly black on stemmaticum and metasoma white to light brown (Fig. 1a). Palpi and spurs white. Antenna brown except scape and pedicel yellow-brown, first three to four flagellomeres white. Legs pale yellow basally to brown on apical 2/3 of hind tibia and most part of tarsi. Wing membranes hyaline, fore wing with pterostigma brown, all veins pale brown.

**Female.** Unknown.

**Host.** Unknown.

#### Material examined.

***Holotype***: • 1♂, Madagascar, Tulear Berenty 12 km, NW Amboasary, JS Noyes, MC Day, 5–15.V.1983, B.M. 1983-201, No. NHMUK010639460.

#### Distribution.

Madagascar.

#### Etymology.

The specific name “*albobasalis*” refers to the white basal three to four flagellomeres. Noun in apposition.

#### Remarks.

This species is similar to *D. rufa* Foerster but differs in the following: penultimate flagellomere 1.4 × longer than wide (subcuboid to cubic in *D. rufa*); punctures on the mesoscutum confluent to strigose posteriorly (not confluent in *D. rufa*); and midlongitudinal carina half the length of its apical bifurcated carina on the propodeum (distinctly longer than its apical bifurcated carina in *D. rufa*).

### ﻿ Dirrhope nixoni

Taxon classificationAnimaliaHymenopteraBraconidae

Liu & Polaszek sp. nov.

0C8AE131-3A93-542B-8664-DE59CCFDC92E

https://zoobank.org/71A37E63-8130-4120-A955-4C358683F0A2

Fig. 2

#### Diagnosis.

**Figure 2.**
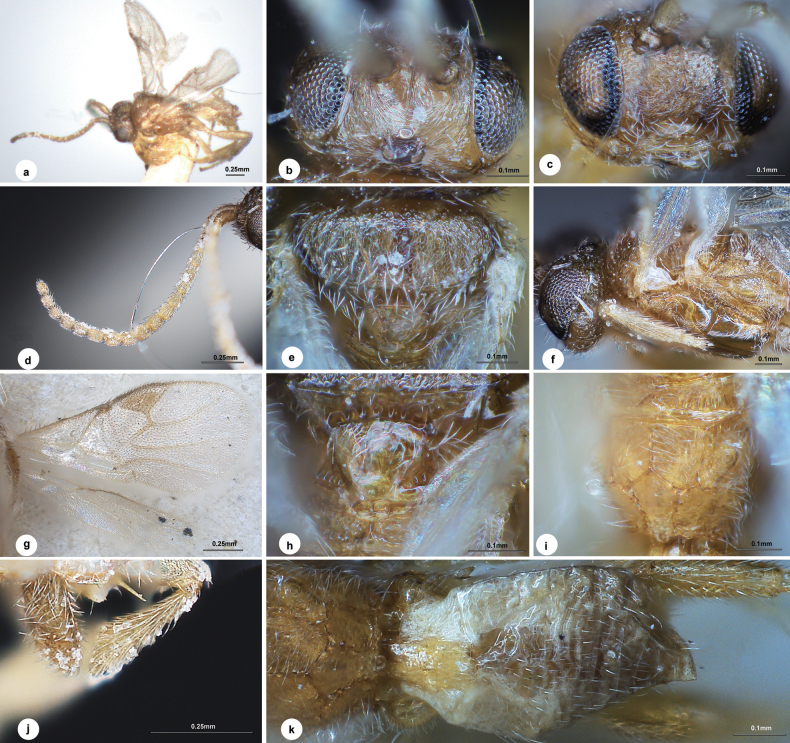
*Dirrhope nixoni* Liu & Polaszek, sp. nov., female. a. Habitus, lateral aspect; b. Head dorsal aspect; c. Head, anterior aspect; d. Antenna; e. Mesosoma, dorsal aspect; f. Mesosoma, lateral aspect; g. Wings; h. Scutellum; i. Propodeum; j. Ovipositor sheath; k. Metasoma, dorsal aspect.

Body length 1.6 mm, mostly light yellow-brown, except head brown, stemmaticum dark brown, T1 yellow, and lateral part of T1–T3 white (Fig. 2a, 2k); eyes 3.0 × longer than temple in dorsal view (Fig. 2b); temple, vertex and frons slightly shiny with numerous strong curved carinae radiated from stemmaticum; temple slightly constricted behind eyes in dorsal view; POL:OD:OOL = 1.3:1.0:3.9; face (Fig. 2c) strigose, 1.4 × wider than high; antenna (Fig. 2d) slightly shorter than body length, 18-merous, with penultimate flagellomere 1.2 × longer than wide, loosely articulated; mesoscutum (Fig. 2e) densely rugose-punctate all over, notauli distinct at anterior declivity, absent posteriorly; scutellum weakly rugulose-punctate anteriorly to nearly polished posteriorly, hind depressions large and ovoid, separated by a carina; propodeum (Fig. 2i) basally densely rugulose with midlongitudinal carina 1.3 × length of its apical bifurcated carina; mesopleuron (Fig. 2f) largely polished; pterostigma (Fig. 2g) 2.2 × as long as its maximum width; vein 1-R1 as long as length of pterostigma; vein r nearly half the length of maximum width of pterostigma, 0.4 × 2-SR; sclerotised part of 3-SR 4.8 × as long as r; vein m-cu 1.2 × as long as 2-SR+M; hind femur 3.8 × as long as its maximum width; inner spur of hind tibia 3/5 length of hind basitarsus; T1 (Fig. 2k) 1.7 × longer than its maximum width; T2 subtriangular, uneven without punctation, suture between T2 and T3 indistinct; ovipositor sheath (Fig. 2j) attenuated apically, 0.3 × as long as hind basitarsus, with short and dense setae entirely.

#### Description.

**Female.** Body length 1.6 mm, fore wing length 1.2 mm (Fig. 2a).

***Head*.** 2.1 × as wide as long, 1.2 × wider than mesoscutum. Eyes 3.0 × longer than temple in dorsal view (Fig. 2b). Temple, vertex and frons slightly shiny with numerous strong curved carinae radiated from stemmaticum. Temple slightly constricted behind eyes in dorsal view. Ocelli small, distance between fore and a hind ocellus 1.3 × longer than minor axis of a hind ocellus, POL:OD:OOL = 1.3:1.0:3.9. Frons flat with a median carina passing between antenna sockets to middle of face. Face (Fig. 2c) shiny, strigose, transverse, 1.4 × wider than high. Clypeus 2.0 × wider than medial length, transversely rugulose laterally. Length of malar space 1.2 × width of mandible. Antenna (Fig. 2d) slightly shorter than body length, 18-merous, with scape, pedicel and 1^st^, 2^nd^, penultimate and ultimate flagellomeres 2.3, 1.3, 2.1, 1.4, 1.2 and 1.3 × longer than wide, 1^st^ 1.2 × longer than 2^nd^, flagellomeres gradually shortened to penultimate flagellomere, loosely articulated.

***Mesosoma*.** Length:width:height = 1.4:1.0:1.0. Mesoscutum (Fig. 2e) shiny, densely rugose-punctate all over, notauli distinct at anterior declivity, absent posteriorly. Scutellar sulcus wide with sparse carinae inside. Scutellum shiny (Fig. 2h), weakly rugulose-punctate anteriorly to nearly polished posteriorly, hind depressions large and ovoid, separated by a carina. Propodeum (Fig. 2i) shiny, basally densely rugulose with one midlongitudinal carina 1.3 × length of its apical bifurcated carina, a broad pentagonal areola present posteriorly, remaining parts uneven. Mesopleuron (Fig. 2f) largely polished. Prepectal carina distinct.

***Wings*.** Fore wing (Fig. 2g): pterostigma wide, 2.2 × as long as its maximum width; vein 1-R1 as long as length of pterostigma; vein r arising from middle of pterostigma, nearly half the length of maximum width of pterostigma, 0.4 × 2-SR; sclerotised part of 3-SR 4.8 × as long as r, 1.8 × 2-SR; vein m-cu 1.2 × as long as 2-SR+M, 0.4 × 2-SR; vein 1-CU1 0.8 × length of cu-a. Hind wing (Fig. 2g): vein M+CU:1-M:r-m = 2.5:1.7:1.0.

***Legs*.** Hind femur 3.8 × as long as its maximum width. Hind tibia widened apically, its length 4.2 × maximum width, 1.1 × length of hind femur. Inner spur of hind tibia 3/5 length of hind basitarsus. Hind basitarsus 0.8 × as long as tarsomeres 2–5.

***Metasoma*.** 0.8 × length of mesosoma. T1 (Fig. 2k) narrow, weakly rugulose, indistinctly broadened before constricted at apical 1/4, 1.7 × longer than its maximum width. T2 subtriangular, uneven without punctation, suture between T2 and T3 indistinct. Hypopygium not exceeding apex of metasoma. Ovipositor sheath (Fig. 2j) very short, attenuated apically, 0.3 × as long as hind basitarsus, with short and dense setae entirely.

***Colour*.** Light yellow-brown, except head brown, stemmaticum dark brown, T1 yellow, and lateral part of T1–T3 white (Fig. 2a, k). Palpi and spurs pale yellow. Antenna yellow-brown except scape and pedicel slightly darker. Ovipositor sheath brown. Legs yellow-brown. Wing membranes hyaline, fore wing with pterostigma brown, vein 1-M, r and 2-SR brown, other veins pale brown.

**Male.** Unknown.

**Host.** Unknown.

#### Material examined.

***Holotype***: • 1♀, South Africa, Port St. John, Pondoland, RE Turner, 15–31.VIII.1923, Brit. Mus 1923-493, det. *Dirrhope* sp. GEJ Nixon, 1963, No. NHMUK010639270. ***Paratype***: • 1♀, same data except 6–25.II.1924, Brit. Mus 1924-136, No. NHMUK010639468.

#### Distribution.

South Africa.

#### Etymology.

The specific name “*nixoni*” is in memory of Dr Gilbert E.J. Nixon who recorded this genus from the Afrotropical region. Noun in the genitive case.

#### Remarks.

This species is similar to *D. ocellimacula* Liu & Polaszek, sp. nov., but differs in the following: antenna 18-merous (19-merous in *D. ocellimacula*); temple distinctly shorter and constricted behind the eyes in dorsal view (slightly shorter and slightly bulged in *D. ocellimacula*); and midlongitudinal carina 1.3 × length of its apical bifurcated carina on the propodeum (0.9 × in *D. ocellimacula*).

### ﻿ Dirrhope ocellimacula

Taxon classificationAnimaliaHymenopteraBraconidae

Liu & Polaszek sp. nov.

438B1454-A54B-5A54-BCA5-98F9DD1EC8FB

https://zoobank.org/F561A40E-5485-48A4-8068-D4C9A98C695E

Fig. 3

#### Diagnosis.

**Figure 3.**
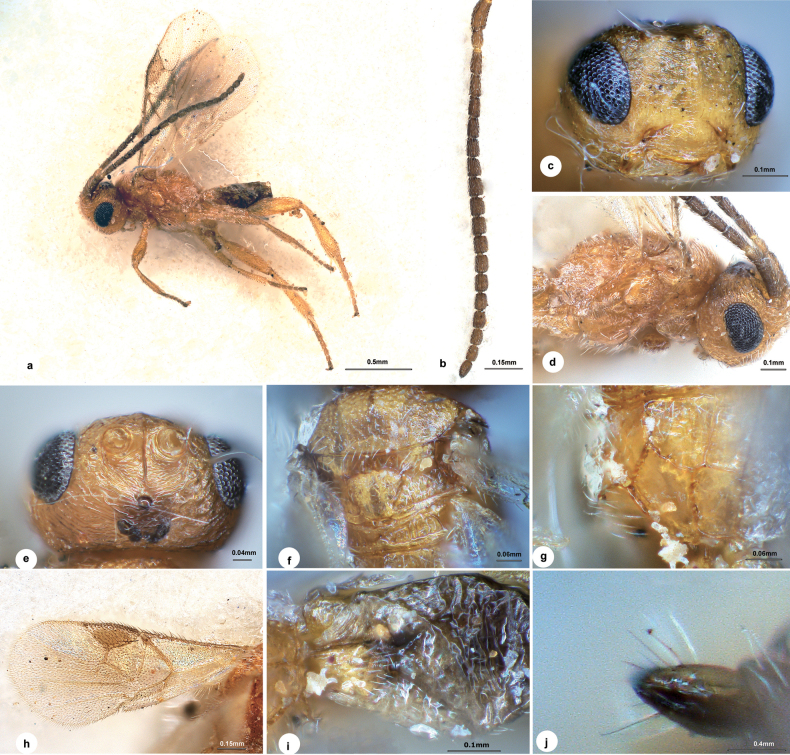
*Dirrhope ocellimacula* Liu & Polaszek, sp. nov., female. a. Habitus, lateral aspect; b. Antenna; c. Head, anterior aspect; d. Mesosoma, lateral aspect; e. Head, dorsal aspect; f. Mesosoma, dorsal aspect; g. Propodeum; h. Wings; i. Metasoma, dorsal aspect; j. Ovipositor sheath.

Body length 1.5 mm, light yellow-brown, except most of metasoma and stemmaticum dark brown to black (Fig. 3a, e, i); eyes 1.7 × longer than temple in dorsal view (Fig. 3e); temple, vertex and frons with strong curved carinae radiated from stemmaticum; temple slightly bulged behind eyes in dorsal view; POL:OD:OOL = 2.1:1.0:4.7; frons with a median carina passing between antenna sockets to less distinct in the half way of face; face (Fig. 3c) weakly strigose, 1.4 × wider than high; antenna (Fig. 3b) 19-merous, with penultimate flagellomere 1.4 × longer than wide; mesoscutum (Fig. 3f) with notauli obvious, extending to scutellar sulcus; scutellum weakly rugose-punctate; propodeum (Fig. 3g) basally slightly rugulose with one midlongitudinal carina 0.9 × length of its apical bifurcated carina; mesopleuron (Fig. 3d) largely longitudinally strigose medially; pterostigma (Fig. 3h) 2.1 × as long as its maximum width; vein 1-R1 0.8 length of pterostigma; vein r half the length of maximum width of pterostigma, 0.4 × 2-SR; sclerotised part of 3-SR 1.5 × as long as r; vein m-cu 1.9 × as long as 2-SR+M; hind femur 3.2 × as long as its maximum width; inner spur of hind tibia 3/5 length of hind basitarsus; T1 1.6 × longer than its maximum width; T2 subtriangular, smooth with shallow punctures, suture between T2 and T3 indistinct; ovipositor sheath (Fig. 3j) 0.3 × as long as hind basitarsus, attenuated apically.

#### Description.

**Female.** Body length 1.5 mm, fore wing length 1.3 mm (Fig. 3a).

***Head*.** 1.9 × as wide as long, 1.2 × wider than mesoscutum. Eyes 1.7 × longer than temple in dorsal view (Fig. 3e). Temple, vertex and frons slightly shiny with numerous strong curved carinae radiated from stemmaticum. Temple slightly bulged behind eyes in dorsal view. Ocelli small, distance between fore and a hind ocellus 1.5 × longer than minor axis of a hind ocellus, POL:OD:OOL = 2.1:1.0:4.7. Frons flat with a median carina passing between antenna sockets to less distinct in the half way of face. Face (Fig. 3c) shiny, weakly strigose, transverse, 1.4 × wider than high. Clypeus 1.8 × wider than medial length, rugulose. Length of malar space nearly as long as width of mandible. Antenna (Fig. 3b) indistinctly longer than body length, 19-merous, with scape, pedicel and 1^st^, 2^nd^, penultimate and ultimate flagellomeres 2.0, 1.4, 1.9, 1.8, 1.4 and 1.5 × longer than wide, 1^st^ 1.2 × longer than 2^nd^, flagellomeres gradually shortened to penultimate flagellomere, loosely articulated.

***Mesosoma*.** Length:width:height = 1.8:1.0:1.3. Mesoscutum (Fig. 3f) shiny, rugose-punctate all over, notauli obvious, extending to scutellar sulcus. Scutellar sulcus wide with sparse carinae inside. Scutellum shiny, weakly rugose-punctate especially anteriorly and laterally, hind depressions large and oblong, separated by a carina. Propodeum (Fig. 3g) shiny, basally slightly rugulose with one midlongitudinal carina 0.9 × length of its apical bifurcated carina, a broad pentagonal areola present posteriorly, remaining parts smooth. Mesopleuron (Fig. 3d) largely longitudinally strigose medially. Prepectal carina distinct.

***Wings*.** Fore wing (Fig. 3h): pterostigma wide, 2.1 × as long as its maximum width; vein 1-R1 0.8 length of pterostigma; vein r arising from middle of pterostigma, half the length of maximum width of pterostigma, 0.4 × 2-SR; sclerotised part of 3-SR 1.5 × as long as r; vein m-cu 1.9 × as long as 2-SR+M, 0.4 × 2-SR; vein 1-CU1 1.3 × length of cu-a. Hind wing (Fig. 3h): vein M+CU:1-M:r-m = 2.3:1.4:1.0.

***Legs*.** Hind femur 3.2 × as long as its maximum width. Hind tibia strongly widened apically, its length 4.1 × maximum width, 1.1 × length of hind femur. Inner spur of hind tibia 3/5 length of hind basitarsus. Hind basitarsus 0.9 × as long as tarsomeres 2–5.

***Metasoma*.** 0.8 × length of mesosoma. T1 (Fig. 3i) narrow, weakly rugulose, indistinctly broadened before constricted at apical 1/4, 1.6 × longer than its maximum width. T2 subtriangular, smooth with shallow punctures, suture between T2 and T3 indistinct. Hypopygium not exceeding apex of metasoma. Ovipositor sheath (Fig. 3j) very short, attenuated apically, 0.3 × as long as hind basitarsus, with long and sparse setae apically.

***Colour*.** Light yellow-brown, except most of metasoma dark brown to black and stemmaticum dark brown with two black maculae behind hind ocelli (Fig. 3a, e, i). Palpi and spurs pale yellow. Antenna dark brown except scape and pedicel yellow-brown. Ovipositor sheath black-brown. Legs yellow. Wing membranes hyaline, fore wing with pterostigma dark brown, vein 1-M, r, 2-SR, 1-CU1 and cu-a dark brown, other veins brown to pale brown.

**Male.** Unknown.

**Host.** Unknown.

#### Material examined.

***Holotype***: • 1♀, South Africa, Cape Province, Mossel Bay, RE Turner, V.1921, Brit. Mus 1921-248, det. *Dirrhope* sp. GEJ Nixon, 1963, No. NHMUK010639399.

#### Distribution.

South Africa.

#### Etymology.

The specific name “*ocellimacula*” refers to the black maculae behind the hind ocelli. Noun in apposition.

#### Remarks.

This species is similar to *D. indica* Ranjith in having no sublateral cells near the areola on the propodeum and with a distinct midlongitudinal carina on the face, but differs in the following: notauli completely extending to the scutellar sulcus (notauli absent posteriorly in *D. indica*); mesopleuron largely longitudinally strigose medially (largely polished in *D. indica*); and the propodeum with the midlongitudinal carina 0.9 × its apical bifurcated carina (1.2 × longer in *D. indica*).

## Supplementary Material

XML Treatment for Dirrhope

XML Treatment for Dirrhope albobasalis

XML Treatment for Dirrhope nixoni

XML Treatment for Dirrhope ocellimacula

## ﻿Additional information

### Use of AI

No use of AI was reported.

### Author ORCIDs

Zhen Liu https://orcid.org/0000-0002-8670-0205

Andrew Polaszek https://orcid.org/0000-0002-7171-3353

### Data availability

All of the data that support the findings of this study are available in the main text.
